# Cyclin Y Is Expressed in Platelets and Modulates Integrin Outside-in Signaling

**DOI:** 10.3390/ijms21218239

**Published:** 2020-11-03

**Authors:** Anastasia Kyselova, Mauro Siragusa, Julian Anthes, Fiorella Andrea Solari, Stefan Loroch, René P. Zahedi, Ulrich Walter, Ingrid Fleming, Voahanginirina Randriamboavonjy

**Affiliations:** 1 Institute for Vascular Signaling, Centre of Molecular Medicine, Goethe University, Frankfurt am Main, 60590 Frankfurt, Germany; kyselova@vrc.uni-frankfurt.de (A.K.); Siragusa@vrc.uni-frankfurt.de (M.S.); JulianAnthes94@gmx.de (J.A.); Fleming@vrc.uni-frankfurt.de (I.F.); 2 German Center of Cardiovascular Research (DZHK), Partner site Rhein Main, 17475 Greifswald, Germany; stefan.loroch@isas.de (S.L.); Rene.Zahedi@ladydavis.ca (R.P.Z.); Ulrich.walter@unimedizin-mainz.de (U.W.); 3 Leibniz–Institute for Analytical Sciences (ISAS)- e.V., Otto-Hahn-Str. 6b, 44227 Dortmund, Germany; fiorella.solari@isas.de; 4 Center for Thrombosis and Hemostasis (CTH), University Medical Center Mainz, 55131 Mainz, Germany

**Keywords:** cyclin Y, integrin, outside-in signaling, spreading

## Abstract

Diabetes is associated with platelet hyper-reactivity and enhanced risk of thrombosis development. Here we compared protein expression in platelets from healthy donors and diabetic patients to identify differentially expressed proteins and their possible function in platelet activation. Mass spectrometry analyses identified cyclin Y (CCNY) in platelets and its reduced expression in platelets from diabetic patients, a phenomenon that could be attributed to the increased activity of calpains. To determine the role of CCNY in platelets, mice globally lacking the protein were studied. *CCNY*^-/-^ mice demonstrated lower numbers of circulating platelets but platelet responsiveness to thrombin and a thromboxane A_2_ analogue were comparable with that of wild-type mice, as was agonist-induced α and dense granule secretion. CCNY-deficient platelets demonstrated enhanced adhesion to fibronectin and collagen as well as an attenuated spreading and clot retraction, indicating an alteration in “outside in” integrin signalling. This phenotype was accompanied by a significant reduction in the agonist-induced tyrosine phosphorylation of β3 integrin. Taken together we have shown that CCNY is present in anucleated platelets where it is involved in the regulation of integrin-mediated outside in signalling associated with thrombin stimulation.

## 1. Introduction

During physiological hemostasis, platelets are activated and are recruited to sites of vascular injury where they adhere and aggregate. However, under pathological conditions abnormal platelet activation can increase the risk of thrombosis development. Although the regulation of hemostasis and thrombosis are the primary role attributed to platelets, there is increasing evidence demonstrating their involvement in different physiological processes including inflammatory reactions, angiogenesis, cancer progression and metastasis [1,2]. Indeed, platelets store a variety of bioactive molecules in their granules and express different receptors on their surfaces conferring them diverse functions.

Despite being anucleated, platelets are known to express mRNA and a plethora of microRNAs and are able to de novo synthesize proteins [3,4,5]. There have been some surprises in the platelet proteome including the transcription factors nuclear factor κB (NFκB) and Peroxisome proliferator-activated receptor gamma (PPARγ) that play non-genomic roles in platelets [6]. Platelets also express functional alpha7-nicotinic acetylcholine receptor [7] and more recently functional ectonucleotidases [8] and collagen [9] have been added to the list of proteins expressed in platelets. Given that platelet proteome is changed upon platelet activation [10] and during different pathological conditions [11,12], a comparison of the proteome of platelets from healthy individuals and from patients would extend our current understanding of platelet activation.

Diabetes is associated with changes in platelet activation [13]. Indeed, platelets from patients with diabetes are hyper-reactive to certain stimuli and they demonstrate increased thrombin-induced adhesiveness, aggregation, degranulation and thrombus formation. One of the mechanisms known to be responsible of diabetes-associated platelet hyperactivation is the activation of the calcium-dependent proteases or calpains, that modify protein function via limited proteolysis [14,15]. The aim of the present study was to identify platelet proteins altered by diabetes.

## 2. Results

### 2.1. Cyclin Y Is Expressed in Platelets and Is Ubiquitinated in Response to Platelet Stimulation by Thrombin

**Figure 1:**
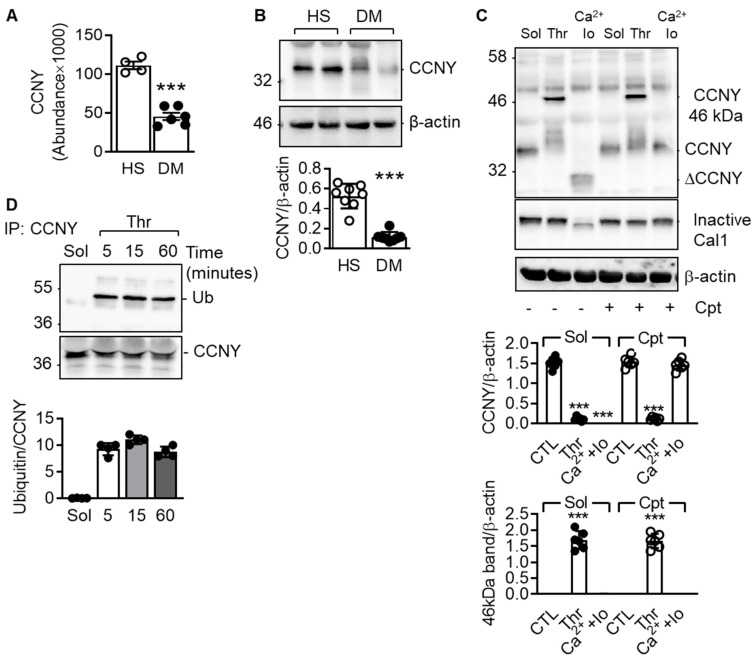
CCNY is expressed in human platelets and is monoubiquitinated upon platelet activation. (**A**) Graph showing levels of CCNY measured by mass spectrometry in platelets from healthy donors (HS) and from diabetic patients (DM); *n =* 4–6 (Student’s *t* test); (**B**) Blot showing the levels of CCNY in platelets from healthy donors (HS) and from diabetic patients (DM); *n =* 8 (Student’s *t* test); (**C**) Representative blots showing the effect of thrombin (1 U/mL, 10 min) or Ca^2+^ (5 mmol/L) and ionomycin (Io,1 µmol/L), in the absence or in the presence of the calpain inhibitor calpeptin (cpt, 10 µmol/L) on the levels of CCNY in washed human platelets; *n =* 6 (ANOVA and Newman-Keuls post-test); (**D**) CCNY was immunoprecipitated (IP) from platelets stimulated with either solvent (sol) or with thrombin (Thr, 1 U/mL) and immunoblotted with an antibody against ubiquitin (Ub) and CCNY; *n* = 4. *** *p* < 0.001.

To identify proteins differentially expressed in platelets from healthy individuals versus patients with type 2 diabetes, platelet lysates were subjected to mass spectrometry. A total of 1355 proteins were quantified with at least two unique peptides (Table S1). After averaging the ratios among the groups and correcting P values, cyclin Y (CCNY) was the only protein that was found to be significantly differentially expressed and significantly lower in platelets from diabetic patients. (Figure 1A). CCNY is a membrane associated cyclin that binds to and activates the cyclin-dependent kinase 14 (CDK14) and given that it was not known to be expressed in platelets, we focussed on this protein for subsequent analyses. The loss of CCNY expression in platelets from diabetic individuals was confirmed by Western blotting (Figure 1B).

The first issue addressed was the reason for the loss of the protein in diabetes. CCNY was identified as a calpain target as the stimulation of washed platelets from healthy donors with the combination of Ca^2+^ and ionomycin, which we have shown to activate calpain [14], led to the disappearance of the full-length protein and the appearance of a 20 kD cleavage product (Figure 1C). As this effect was not observed in the presence of the calpain inhibitor calpeptin, the loss of CCNY in diabetic platelets was most likely the consequence of calpain activation. The activation of Ca^2+^ -activated cysteine proteinases (calpains) in platelets from diabetic patients has been reported to result in the limited proteolysis of some proteins [15]. Interestingly, thrombin, which was included as a control in the latter experiments as it does not activate calpain, led to an apparent increase in the molecular mass of CCNY (shift from 36 to 46 kD). As expected, the thrombin-induced shift in CCNY was not affected by calpain inhibition.

Post translational modifications can cause such an effect and as cyclin family proteins are known to be ubiquitinated in different cell types [16,17,18], we focussed on determining whether or not CCNY was ubiquitinated in response to thrombin stimulation. Indeed, while the CCNY immunoprecipitated from solvent-treated platelet lysates was not recognized by anti-ubiquitin antibodies, a clear signal was detected in thrombin-stimulated platelets (Figure 1D). This response was specific to thrombin and accompanied by a change in the detergent solubility of CCNY since neither the thromboxane analogue, U46619, nor ADP elicited CCNY ubiquitination in the absence or presence of the Ubiquitin-activating enzyme (E1) inhibitor; NSC 624206 (Figure S1).

### 2.2. Cyclin Y Is Involved in Platelet Biogenesis and Function

**Figure 2:**
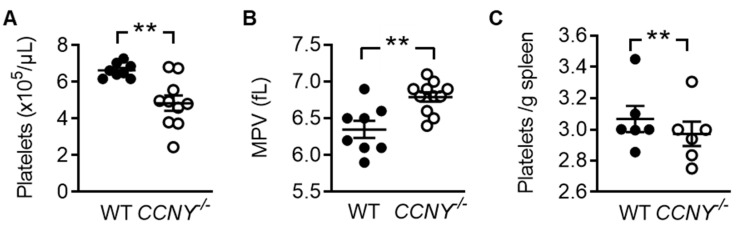
CCNY is important for platelet biogenesis. (**A**) Number of platelets in whole blood from wildtype (WT) vs *CCNY*^-/-^ mice; (**B**) Mean platelet volume in wildtype (WT) vs *CCNY*^-/-^ mice; (**C**) Number of platelets in the spleen from wildtype (WT) vs *CCNY*^-/-^ mice. *n* = 6–10 (Student’s t test), ** p < 0.01.

To characterize the function of CCNY in platelets, mice globally lacking the protein (*CCNY*^-/-^ mice) were generated. As reported previously [19], *CCNY*^-/-^ mice are viable but both genders demonstrated a reduced body weight (Figure S2). However, although blood counts were similar in wild-type and *CCNY*^-/-^ mice (Table S2), the deletion of CCNY resulted in a significant decrease in platelet number (Figure 2A) and an increase in mean platelet volume (Figure 2B). To assess whether the low platelet count was the consequence of enhanced clearance, platelet numbers in the spleens from the two genotypes were compared. No differences were detected suggesting that the reduced platelet count is more likely to reflect a defect in platelet biogenesis (Figure 2C). *CCNY*^-/-^ mice also demonstrated an enhanced bleeding time (74 ± 10 s in wild-type vs 126 ± 22 s in *CCNY*^-/- -/-^ mice, *n* = 15, *p* = 0.041).

**Figure 3:**
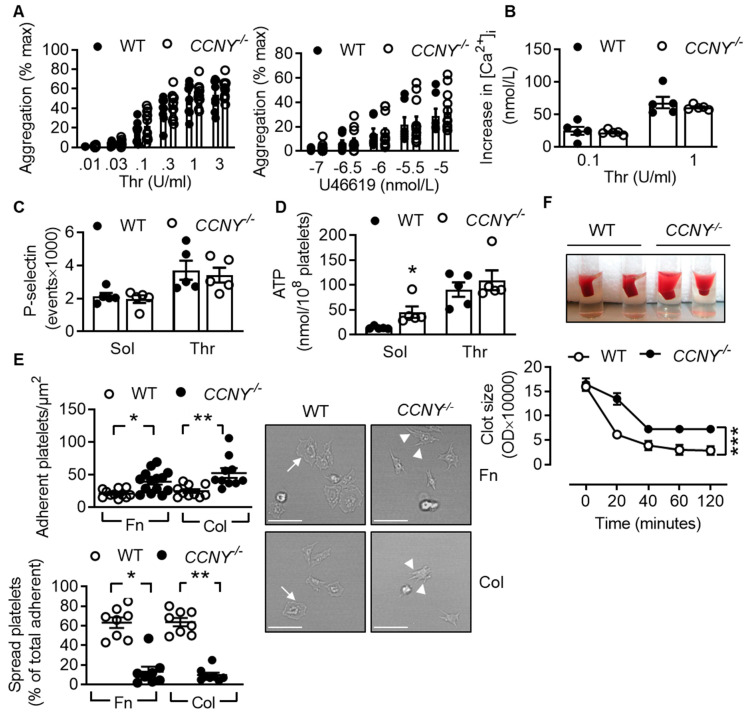
CCNY is important for platelet spreading but dispensable for platelet aggregation, change in intracellular Ca^2+^ concentration and degranulation: (**A**) Aggregation induced by thrombin (Thr) or by the thromboxane A2 analogue U46619 of platelets from wildtype (WT) and *CCNY*^-/-^ mice; *n* = 7–12; (**B**) Increase in intracellular calcium levels in washed platelets from wildtype (WT) and CCNY^-/-^ mice after stimulation with thrombin (Thr); *n* = 5; (**C**) Flow cytometry analysis of the expression of P-selectin on the surface of washed platelets from wildtype (WT) and *CCNY*^-/-^ stimulated or not with thrombin (Thr, 1 U/mL); *n* = 5; (**D**) Levels of ATP measured in the releasate from platelets stimulated with either solvent (Sol) or thrombin (Thr, 1 U/mL); *n* = 5; (**E**) Representative images and graphs showing levels of adherent and spread platelets from wild-type (WT) and *CCNY*^-/-^ mice on collagen (Col) or fibronectin (Fn); arrows represent lamellipodia, arrow heads show filopodia; bar = 10 µm; *n* = 11–14; (F) Clot retraction of platelet-rich plasma from wild-type (WT) and *CCNY*^-/-^ mice after stimulation with thrombin (1 U/mL); *n* = 6 (ANOVA and Newman-Keuls post-test). * *p* < 0.05 ** *p* < 0.01 *** *p* < 0.001.

Next, we compared the function of platelets from wild-type and *CCNY*^-/-^ mice. When platelet numbers were adjusted to similar values, the aggregation induced by thrombin and the thromboxane A_2_ analogue were comparable (Figure 3A). There was also no difference in the Ca^2+^ response to thrombin between the genotypes (Figure 3B) or in the secretion of α granule contents (Figure 3C). Interestingly, the basal release of dense granule contents was significantly higher in platelets from *CCNY*^-/-^ compared to wild-type platelets whereas similar levels were released after agonist stimulation (Figure 3D). There were, however, clear differences in the ability of platelets from wild-type and *CCNY*^-/-^ mice to adhere and spread. Even though more platelets from *CCNY*^-/-^ mice adhered to collagen and fibronectin, their spreading was impaired and although CCNY-deficient platelets formed filopodia they failed to generate lamellipodia (Figure 3E). A clear delay in clot retraction was also evident in platelet-rich plasma from *CCNY*^-/-^ versus wild-type mice (Figure 3F), suggesting an alteration of the outside in signalling.

### 2.3. CCNY Regulates β3 Integrin Tyrosine Phosphorylation

**Figure 4:**
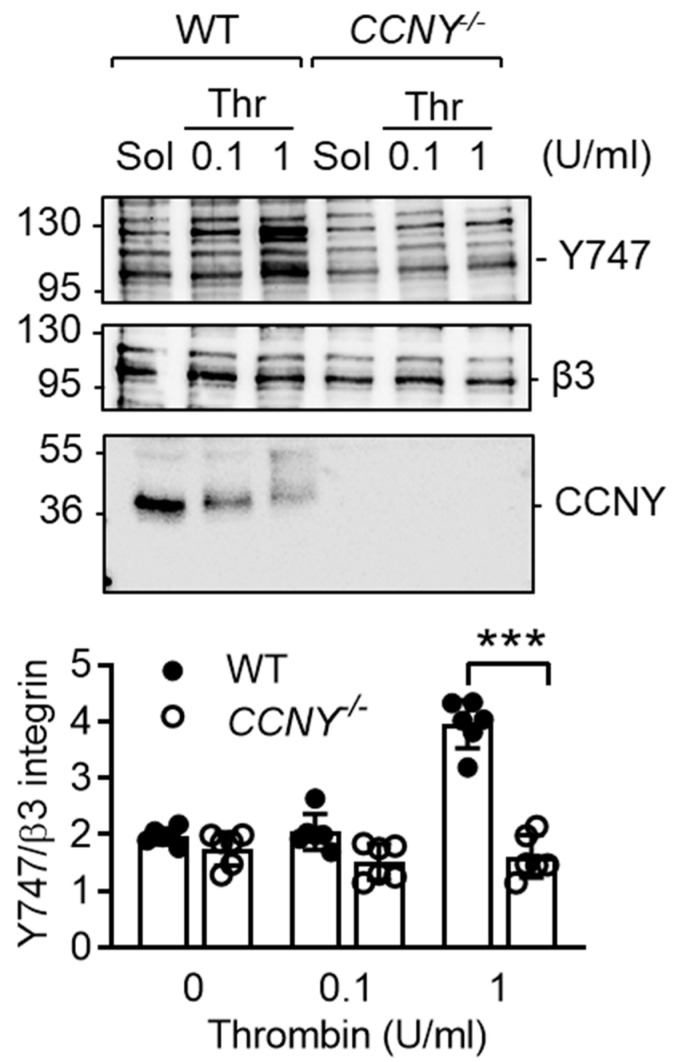
CCNY is important for β3-integrin-mediated outside-in signaling: Representative blots and graph showing the phosphorylation of β3-integrin in platelets from wild-type (WT) and *CCNY^-/-^* mice stimulated with either solvent (Sol) or thrombin (Thr, 0,1 and 1 U/mL); *n* = 6 (ANOVA and Newman-Keuls post-test. *** *p* < 0.001.

The activation of β3 integrin and its phosphorylation on Tyr747 is an essential part of outside-in signalling [20] and while thrombin elicited the phosphorylation of β3 integrin in platelets from wild-type mice, significantly lower phosphorylation was detected in *CCNY*^-/-^ platelets (Figure 4). 

## 3. Discussion

**Figure 5:**
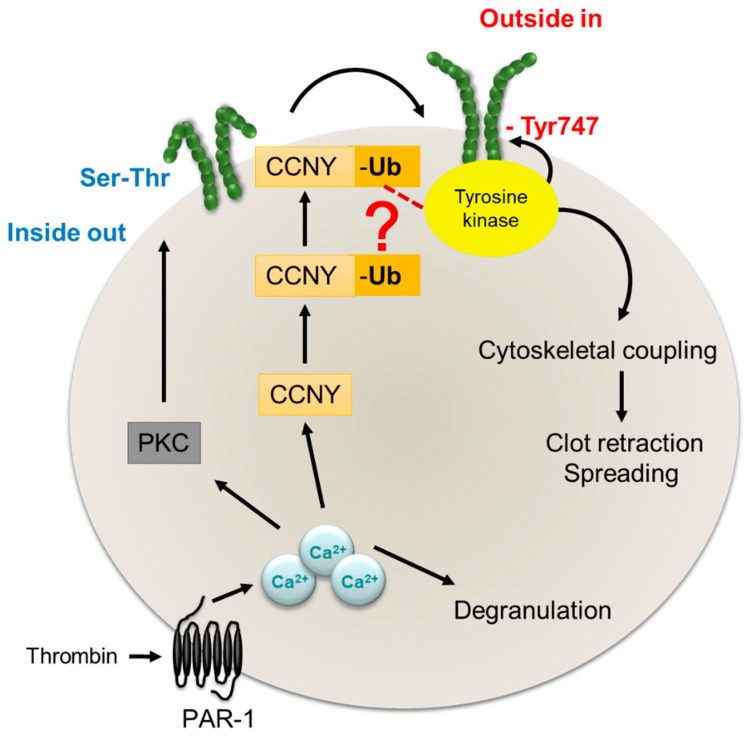
Role of CCNY in β3-integrin-mediated outside-in signaling: CCNY is expressed in human and murine platelets. Stimulation of platelets with thrombin leads to the mono-ubiquitination of CCNY and its translocation to the plasma membrane where it regulates β3 integrin tyrosine phosphorylation and outside-in signaling. Arrows represent defined processes and dotted arrow shows possible mechanism.

The present study identified CCNY in human and murine platelets and reports a role for this protein in the regulation of β3 integrin outside-in signalling. We found that upon thrombin stimulation, CCNY is ubiquinated and very likely translocates to the plasma membrane to regulate the tyrosine phosphorylation of β3 integrin (Figure 5). The loss of CCNY results in the alteration of platelet biogenesis and platelet spreading as well as an increased bleeding.

There is increasing evidence that platelets express proteins that were initially thought to be only present in nucleated cells – good examples being the transcription factors PPARγ and NFκB [21,22]. Clearly, these proteins play non transcriptional roles in platelets as PPARγ was shown to attenuate platelet activation by preventing the surface expression and release of CD40L and thromboxane B(2) whereas NFκB forms a complex with protein kinase A. The release of PKA upon platelet activation was demonstrated as a novel feedback inhibitory mechanism to modulate platelet functions.

In the present study, CCNY was discovered by a proteomic study that was initially aimed to identify proteins that are differentially expressed in platelets from healthy donors and from patients with type 2 diabetes. CCNY is one of the most highly conserved members of the cyclin superfamily of proteins and was originally identified as a protein that interacts with cyclin-dependent kinase 14, and is involved in cell cycle and transcription regulation. [23]. Different functions have been attributed to CCNY including the regulation of cancer cell proliferation [24,25], Wnt signalling [26,27] as well as the control of adipogenesis [19]. However, CCNY has also been detected in non-dividing neuronal cells where it is thought to play a variety of roles that are independent of cell cycle and proliferation [28,29,30]. Certainly, CCNY is involved in targeting presynaptic components to the axon and in synapse elimination during development. The function of CCNY in platelets was characterized in *CCNY^-/-^* mice, which demonstrated a marked depression in platelet biogenesis suggesting that in megakaryocytes CCNY might be involved in proplatelet formation. Indeed, just such a function has been reported for Cyclin D1 [31]. 

The most impressive phenotype of *CCNY^-/-^* platelets was the defect in spreading, a complex process that involves integrin phosphorylation and activation. Among the plethora of integrins expressed by platelets, the α_IIb_β_3_ integrin complex represents the majority of platelet integrin [32] with approximately 80,000 copies expressed on the surface of unstimulated platelets [33]. Upon platelet activation, the serine/threonine phosphorylation of the intracellular tail of β_3_ integrin and conformational changes leads to its activation (inside-out signaling). The active α_IIb_β_3_ can bind different platelet agonists including fibrinogen, vWF and fibronectin that initiates intracellular signaling i.e., tyrosine phosphorylation of the intracellular tail of the β_3_ integrin and the recruitment of kindlin 3 (outside-in signaling). The Src-family kinase Fyn is the tyrosine kinase responsible for the tyrosine phosphorylation of β_3_ integrin in platelets [34]. In the absence of CCNY the tyrosine phosphorylation of β_3_ integrin was altered, suggesting a crucial role for CCNY in the regulation of β_3_ integrin activity. Given that following thrombin stimulation, CCNY was ubiquitinated, a modification that was concomitant with a change in the solubility of the protein, it seems that thrombin elicits the translocation of CCNY to the platelet membrane to affect β_3_ integrin function Indeed, contrary to polyubiquitination that targets proteins to proteasomal degradation [35], monoubiquitination localizes proteins to specific cellular compartments [36] and has been reported to regulate protein complex formation [37]. Currently, it is not known whether CCNY serves as a molecular scaffold that would facilitate the association of a tyrosine kinase with β3 integrin and a detailed characterization of the mechanism will be the subject of a future study. It is also not clear how CCNY becomes ubiquitinated and whether the mechanism described in this study is specific to platelets or also applies to other cells. It was however possible to demonstrate that CCNY levels were lower in platelets from diabetic individuals, as this could be attributed to the activation of Ca^2+^ activated proteases or calpains. This is important as we have previously shown that diabetic mice developed large but unstable thrombus [14], and calpain inhibition has been reported to prevent platelet-hyperreactivity in mouse models of diabetes [14].

## 4. Materials and Methods

### 4.1. Reagents

Fibronectin was from BD transduction laboratories (Heidelberg, Germany). Thrombin was from Hemochrom Diagnostica (Essen, Germany). The anti-cyclin Y antibody and the anti-CD42b were from Abcam (Cambridge, UK). All other compounds were from Sigma-Aldrich (Merck, Darmstadt, Germany).

### 4.2. Study Subjects

A total of nine patients (four women, five men; mean age, 47.11 ± 4.9 years, age range, 20 to 60 years, Hemoglobin (Hb)A1c, 8.57 ± 0.41%; fasting plasma glucose, 155.17 ± 20.72 mg/dL and BMI = 28.68 ± 1.78) with type 2 diabetes mellitus either without treatment or treated with metformin were included in the study. Fifteen age-matched subjects without diabetes or insulin resistance served as the control group (eight women, seven men; mean age, 39.06 ± 3.87, age range, 25 to 63 years). None of the participants took any medication known to interfere with platelet aggregation for at least 10 days before fasting blood sampling. The study protocol was approved by the ethics committee of the Goethe University Hospital (No. E 61/09 Geschäfts Nr 86/09, 9 June 2009) and the Landesärztekammer Hessen. All of the participants gave written informed consent. 

### 4.3. Animals

Mice globally lacking cyclin Y (*CCNY*^-/-^) were generated by Genoway (Lyon, France) using CRISPR/Cas9 technology by inserting a premature Stop codon in exon 1 of *CCNY* gene. *CCNY* heterozygous mice (MGI:1915224) were developed onto a pure C57BL/6N genetic background (Charles River Laboratories, Sulzfeld, Germany) and mice were bred at the Goethe University to generate homozygous knockout animals. All animals were housed in conditions that conform to the Guide for the Care and Use of Laboratory Animals published by the US National Institutes of Health (NIH publication no. 85-23). Both the university animal care committee and the Federal Authorities for Animal Research, Regierungspräsidium Darmstadt (Hessen, Germany) approved the study (study number: Fu-1264, 24^th^ April 2020). 

### 4.4. Blood Counting

Mice were anesthetized with isoflurane and blood was collected via cardiac puncture into EDTA-coated capillary tubes. Samples of whole blood (100 µL) were counted using an automated hematology analyzer VetScan HM5 (Abaxis, Griesheim, Germany).

### 4.5. Flow Cytometry

Spleens were shredded manually and suspended in phosphate-buffered saline. Thereafter, suspension was passed through a 40 µM filter and lysed with red cell lysis buffer and centrifuged (4000 rpm, 10 min). Thereafter, samples were fixed with formaldehyde (2% in PBS *v*/*v*, 15 min), washed and incubated in the dark for 15 min at room temperature with FITC-conjugated anti-CD42 antibody (Emfret Analytics GmbH, Würzburg, Germany) to stain platelets. IgG1-FITC antibody (BD transduction laboratories; Heidelberg, Germany) served as an isotype control. Data were acquired and analyzed using a FACSCalibur flow cytometer (BD Biosciences, Heidelberg, Germany). 

### 4.6. Platelet Isolation

*Human platelets:* Platelets were isolated as described [38], and samples were either directly used for functional assays or lysed for Western blotting or proteomic analyses or snap frozen and stored at −80 °C until use. 

*Murine platelets:* Mice were anesthetized with isoflurane and blood was collected via cardiac puncture into a syringe containing 10% acidic citrate dextrose (120 mmol/L sodium citrate, 110 mmol/L glucose, 80 mmol/L citric acid) as anticoagulant. Platelets were prepared from whole blood by differential centrifugation and resuspended in HEPES buffer as described [14].

### 4.7. Platelet Aggregation

Aggregation of washed murine platelets (2.5 × 10^8^ platelets/mL) was measured using an 8-channel aggregometer (PAP8, Mölab, Langenfeld, Germany).

### 4.8. ATP Assay

The release of ATP was determined using a luciferin/luciferase ATP kit (Enliten ATP assay system; Promega, Walldorf, Germany) as described [39].

### 4.9. P-selectin Expression

Washed platelets from wild-type or *CCNY*^-/-^ mice were stimulated with either solvent or thrombin (1 U/mL, 10 min). After stimulation, platelets were fixed with formaldehyde (2% in PBS *v*/*v*, 15 min), washed and incubated with FITC-conjugated anti-P-selectin antibody, or control mouse IgG for 15 min at room temperature. After washing, surface expression of P-selectin was analyzed using a FACSCalibur flow cytometer (BD Biosciences, Heidelberg, Germany).

### 4.10. Platelet Adhesion and Spreading Assays

Static adhesion assays were performed as described [38]. Suspensions of platelets from wild-type and *CCNY*^-/-^ mice (5 × 10^4^ platelets/µL) were seeded on 8-wells glass µ-slides (ibidi, Martinsried, Germany), coated with either fibronectin (100 µg/mL) or collagen (1.8 ng/mL) and incubated at 37 °C for 60 min. Non-adherent platelets were removed by washing and adherent and spread platelets were fixed. Images were captured by an AxioCam MRm on a Cell Observer microscope (Zeiss, Jena, Germany) and visualized using the imaging software AxioVision 4.8 (Zeiss, Jena, Germany). Platelets were counted and classified according to their shape. Platelets with lamellipodia were considered fully spread and the ones with filopodia were not fully spread. 

### 4.11. Clot Retraction

Platelet-rich plasma obtained by centrifugation of whole blood at 250× *g* for 10 min, was stimulated with 1 U/mL thrombin in the presence of CaCl_2_ (2 mmol/L) and 2 µL erythrocytes to enhance the contrast of the clot. The clots were allowed to retract for up to 3 h at room temperature and were photographed at different times. The extent of retraction was quantified using Bio-1D software (version 15.05, Vilber Lourmat, Eberhardzell, Germany).

### 4.12. Bleeding Time

Mice were restrained in appropriate restrainer and placed on a heated mat and anesthetized with isoflurane (3 to 5%). A 1–2 mm section of the tail tip was cut, and the tail tip was immediately immersed in sterile saline solution at 37 °C. The bleeding time (i.e., the time between initial flow of blood and its cessation) was recorded. When no blood was observed on the saline after 60-second intervals, bleeding was considered to have ceased. The experiment was stopped after 20 min to prevent bleeding.

### 4.13. LC-MS-based Proteomics

For proteomics analysis, purified platelets from 4 healthy donors and 6 diabetic patients were lysed in 50 mmol/L TRIS-HCL, 150 mmol/L NaCl, 1% sodium dodecyl sulfate (SDS), pH 7.8 including one tablet cOmplete Mini and one tablet PhosSTOP (Roche, Basel, Switzerland) per 10 mL. Protein concentrations were determined using the bicinchoninic acid assay (Pierce, Thermo-Fisher Scientific, Bremen, Germany). Afterwards, cysteines were reduced by 30 min incubation at 56 °C with 10 mmol/L dithiothreitol and free sulfhydryl groups were alkylated with 30 mmol/L iodoacetamide for 30 min at RT in the dark. 80 µg of each samples were processed using filter-aided sample preparation (FASP) using 30 kDa molecular weight cut-off (spin-) filters [40,41]. In brief, samples were diluted with 8 mol/L urea in 100 mmol/L TRIS-HCl, pH 8.5 to a final concentration of 0.25 % SDS and loaded onto the filters followed by centrifugation for 25 min (13,500× *g*, as well used for all following steps). The retained protein fraction was washed three times with 100 µL 8 M urea in 100 mmol/L Tris-HCl at pH 8.5 and three times with 100 µL 50 mmol/L ammonium bicarbonate (ABC) followed by 15 min of centrifugation. Digestion was performed in 50 mmol/L ABC, 200 mmol/L guanidine hydrochloride, 2 mmol/L CaCl_2_ using trypsin (Promega, Sequencing Grade Modified, Madison, WI, USA) with an enzyme to sample ratio of 1:20 (*w*/*w*) for 16 h at 37 °C. Peptides were recovered by centrifugation for 25 min and filters were washed with 50 µL 50 mM ABC and 50 µL of pure water for recovering residual peptides. Samples were acidified by adding trifluoroacetic acid (TFA) to a final concentration of 1 % and digestion quality control was performed via a monolithic column-HPLC [42]. 

Nano LC-MS/MS analysis was conducted using a U3000 RSLCnano online-coupled to a Q Exactive HF mass spectrometer (both Thermo Scientific, Bremen, Germany, including employed HPLC columns). Peptides were loaded onto the trap column (Acclaim PepMap100 C18; 100 µm × 2 cm) in 0.1 % TFA at a flow rate of 20 µL/min. After 5 min, the pre-column was switched in line with the main column (Acclaim PepMap100 C18; 75 μm × 50 cm) and peptides were separated using a 120 min binary gradient ranging from 2.5–35 % acetonitrile in presence of 0.1 % formic acid at 60 °C and a flow rate of 250 nL/min. The MS was operated in data dependent acquisition (DDA) mode with survey scans acquired at a resolution of 60,000 followed by 15 MS/MS scans at a resolution of 15,000 (top15). Precursor ions were selected for MS/MS by intensity, isolated in a 1.6 m/z window and subjected to fragmentation by higher energy collision induced dissociation using a normalized collision energy of 27. Automatic gain control target values were set to 106 and 5×104 and the maximum ion injection was set to 120 ms and 250 ms for MS and MS/MS, respectively. Precursor masses were excluded from re-fragmentation for 30 s (dynamic exclusion) and the polysiloxane at m/z 371.1012 was used as internal calibrant [43]. 

### 4.14. Proteomic Data Analysis

Raw-files were imported into Progenesis QI (Nonlinear Dynamics, Newcastle upon Tyne, UK) for ion trace alignment, feature detection and feature mapping. Exported MS/MS were subjected to a database search against the human Uniprot database (www.uniprot.org, December 2013; 20,274 target sequences, decoys reversed) using X!TANDEM Sledgehammer (2013.09.01.1) and OMSSA implemented in SearchGUI 2.2.2 [44]. Search parameters were: enzyme trypsin with a maximum of two missed cleavages, carbamidomethylation of Cys (+57.0214 Da) as fixed and oxidation of Met (+15.9949 Da) as variable modification. MS and MS/MS tolerances were set to 10 ppm and 0.02 Da. 

Search results were combined in PeptideShaker version 1.2.0 [45] and filtered to meet a 1 % false discovery rate (FDR) on the peptide spectrum match level before re-import into the Progenesis QI software. Peptide sequences containing oxidized Met (+15.9949 Da) and pyro-Glu (−17. 0265), as obtained from the second pass of the X!TANDEM, were omitted from further analysis as well as proteins detected with less than two unique peptides. MS raw files, SearchGUI and peptide shaker search results are deposited in the ProteomeXchange repository [46] and can be accessed via the identifier PXD021271 and 10.6019/PXD021271. (reviewers can access the data via user: reviewer00373@ebi.ac.uk, password: HcIdZJx7).

In total 1355 proteins were quantified with at least 2 unique peptides (Table S1). The mean of abundances for each protein was calculated for each group. Thereafter, means from diabetic patients were divided by those from healthy individuals to calculate ratios, which were transformed to log2. P-values were determined using Welch’s *t*-test resulting in 100 candidate proteins. Notably, at a significance level of 0.05, 68 % of the candidates were expected to be false positive, hence, p-values were corrected for multiple testing using the method of Benjamini-Hochberg (q-value).

### 4.15. Activation of Calpain In Vitro

Washed human platelets were stimulated with the combination of CaCl_2_ (5 mmol/L) and ionomycin (1 µmol/L) for 30 min at 37 °C to activate calpain as previously described [14]. Stimulation was stopped by lysing platelet suspension with Triton X-100 lysis buffer.

### 4.16. Western Blotting

Platelets were lysed in Triton X-100 lysis buffer and lysate was subjected to SDS-PAGE followed by immunoblotting as described [47].

### 4.17. Immunoprecipitation

Platelets were lysed in TAP-lysis buffer (1% NP-40, 50 mmol/L Tris pH7.5, 150 mmol/L NaCl, 10 mmol/L NaPPi, 20 mmol/L NaF, 50 mmol/L β-glycerolphosphate, 2 mmol/L Na_3_VO_4_, 10 nmol/L okadaic acid, Protease Inhibitor Mix, PMSF, 10% glycerol) and lysate was incubated overnight at 4 °C with anti-cyclin Y antibody (Abcam, Cambridge, UK). Immunoprecipitates (antibody/antigen complex) were pulled out of the sample using protein G-coupled agarose beads and subjected to immunoblotting with anti-Fyn (Abcam, Cambridge, UK), anti-β3 integrin (Abcam, Cambridge, UK), or anti-ubiquitin (Calbiochem, Merck, Darmstadt, Germany) antibodies.

### 4.18. Statistical Analysis

Data are expressed as mean ± SEM and statistical evaluation was performed using either Student’s t test, one-way analysis of variance (ANOVA) followed by a Newman-Keuls post-test or two-way ANOVA followed by Tukey’s or Sidak’s post-test where appropriate using Prism software (GraphPad 7). Values of *p* < 0.05 were considered statistically significant.

## Supplementary Materials

The following are available online at https://www.mdpi.com/1422-0067/21/21/8239/s1, Figure S1: Representative blot showing the levels of CCNY and ubiquitin in triton soluble vs triton insoluble fraction in resting platelets and after stimulation with thrombin (Thr, 1 U/mL, 10 min), the thromboxane A2 analogue U46619 (U46, 100 nmol/L, 10 min) or ADP (1 μmol/L, 10 min), Figure S2: Body weight of wild-type and Ccny^-/-^male (left panel) and female (right panel)mice; *n* = 4–9 (Unpaired *t*-test),*** *p* < 0.005., Table S1, Table S2: Blood counts in wild-type and Ccny^-/-^mice.
