# Supplementary material for: Cyclin Y Is Expressed in Platelets and Modulates Integrin Outside-in Signaling
Source: Int J Mol Sci. 2020 Nov 3;21(21):8239. doi: 10.3390/ijms21218239 (PMC7662234; doi:10.3390/ijms21218239)
Supplement: Supplementary file 1 [file ijms-21-08239-s001.zip › Supplemental table 2.pdf]

Supplementary table 2: Blood counts in wild-type and $Ccny^{-/-}$ mice

**Parameter**	**Wild-Type**	**$Ccny^{-/-}$**	**P values**
WBCs (10^9/L)	6.6 ± 0.8	7.05 ± 0.4	n.s
Lymphocytes (10^9/L)	5.1 ± 0.9	5.9 ± 0.4	n.s
Monocytes (10^9/L)	0.2 ± 0.1	0.2 ± 0	n.s
Neutrophils (10^9/L)	1.2 ± 0.5	0.8 ± 0.2	n.s
Erythrocytes (10^9/L)	10.5 ± 0.1	10 ± 0.3	n.s
Hemoglobin (g/dL)	16 ± 0.3	16.5 ± 0.3	n.s
Platelets (10^6/L)	649 ± 19.7	484.6 ± 42.4	0.0034
MPV	6.3 ± 0.1	6. 7 ± 0	0.0024
