# Supplementary material for: Cyclin Y Is Expressed in Platelets and Modulates Integrin Outside-in Signaling
Source: Int J Mol Sci. 2020 Nov 3;21(21):8239. doi: 10.3390/ijms21218239 (PMC7662234; doi:10.3390/ijms21218239)
Supplement: Supplementary file 1 [file ijms-21-08239-s001.zip › Supplementary Figures.pdf]

Supplementary Figures

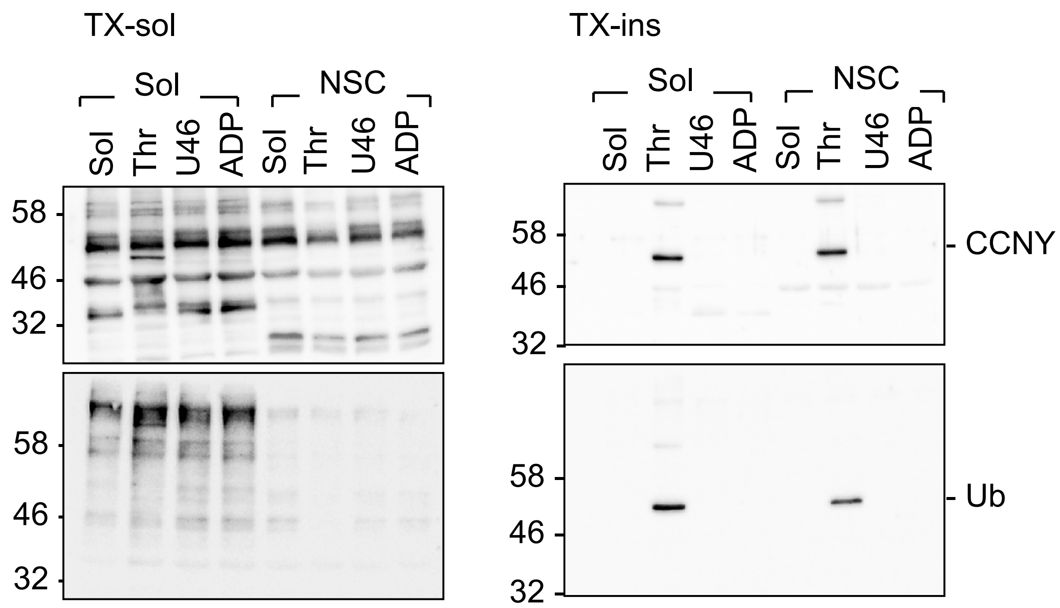

Figure I: Representative blot showing the levels of CCNY and ubiquitin in triton soluble vs triton insoluble fraction in resting platelets and after stimulation with thrombin (Thr, 1U/ml, 10 minutes), the thromboxane A2 analogue U46619 (U46, 100 nmol/L, 10 minutes) or ADP (1 μmol/L, 10 minutes).

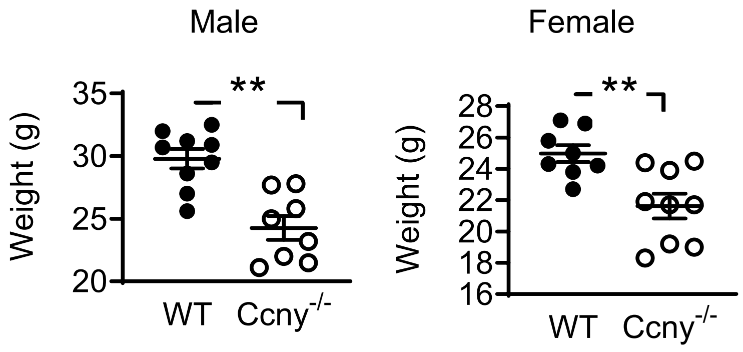

Figure II: Body weight of wild-type and $Ccny^{-/-}$ male (left panel) and female (right panel) mice; n=4-9 (Unpaired *t* test), ***P<0.005.
